# When Full Extension Falls Short: Age and Preoperative Deformity After Persistent Pediatric Trigger Thumb Surgery

**DOI:** 10.3390/jcm15218686

**Published:** 2026-11-07

**Authors:** Mert Karaduman, Caner Öğük, Hakan Şeşen, İsmail Demirkale, Murat Altay

**Affiliations:** 1 Division of Hand Surgery, Department of Orthopaedics and Traumatology, Etlik City Hospital, University of Health Sciences, 06170 Ankara, Türkiye; mertkaraduman@gmail.com; 2 Department of Orthopaedics and Traumatology, Denizli State Hospital, 20150 Denizli, Türkiye; 3 Department of Orthopaedics and Traumatology, Ataturk Sanatoryum Training and Research Hospital, University of Health Sciences, 06290 Ankara, Türkiye; 4 Department of Orthopaedics and Traumatology, Liv Hospital Vadistanbul, 34396 Istanbul, Türkiye

**Keywords:** trigger finger disorder, child, thumb, range of motion, articular, treatment outcome, retrospective studies

## Abstract

**Background:** The optimal timing of surgery for persistent pediatric trigger thumb remains controversial, and evidence regarding clinical factors associated with residual extension deficit after surgery is limited. This study evaluated postoperative improvement in active interphalangeal (IP) joint extension and explored preoperative factors associated with residual active extension deficit. **Methods:** We retrospectively reviewed 26 consecutive patients aged 3–12 years at diagnosis who underwent open A1 pulley release for persistent pediatric trigger thumb between 2012 and 2021. Preoperative and final active IP extension values were extracted from routine clinical notes. Complete correction was defined as active extension to neutral (0°) or beyond, whereas an extension deficit >0° was classified as residual active extension deficit. The last pre-revision assessment was used for the patient who underwent reoperation. Clinical and demographic factors were compared between outcome groups using exact tests on an exploratory basis. **Results:** At a median follow-up of 10 months, complete correction was achieved in 21 patients (80.8%), while five (19.2%) had residual active extension deficit. Four of these five patients had a residual deficit of 1–9°, and one had a deficit of 10–19°. Passive extension restriction accompanied the active deficit in two patients. Patients with residual active extension deficit were older at diagnosis (median, 10.6 vs. 3.8 years) and surgery (median, 10.7 vs. 4.0 years) and had greater preoperative active IP extension deficits (median, 45.0° vs. 20.0°; all *p* < 0.001). The distribution of primary presenting complaints also differed between groups (*p* = 0.009). Four patients with residual deficits reported no ongoing complaints; one underwent revision release at nine months for residual limitation accompanied by functional difficulty. No wound, infectious, or neurovascular complications were recorded. **Conclusions:** Open A1 pulley release resulted in active IP extension to neutral or beyond in most patients with persistent pediatric trigger thumb. Older age and greater preoperative active extension deficit were associated with residual postoperative active extension deficit, although most residual deficits were mild. These associations remain exploratory; their independent prognostic value and clinical significance require confirmation in larger studies with longer follow-up and standardized functional assessment.

## 1. Introduction

Pediatric trigger thumb is a childhood hand condition that may cause limited interphalangeal (IP) joint extension, occasional pain, and functional impairment. Its management remains controversial, particularly regarding treatment strategies and the timing of surgical intervention. The reported incidence is approximately 0.5–3.3 cases per 1000 children, with the thumb being affected substantially more frequently than the other fingers in pediatric triggering conditions [1,2]. Although its etiology remains incompletely understood, a size mismatch between the flexor pollicis longus tendon and the A1 pulley is considered the primary pathophysiological mechanism, and most cases are thought to develop after birth [1,3]. Observation, passive stretching exercises, splinting, and surgical release are the main treatment options. However, there is no consensus regarding the optimal timing of surgical intervention, particularly in children with persistent deformity [2].

A meta-analysis involving 599 thumbs reported a pooled spontaneous resolution rate of 43.5%, while two prospective cohorts reported rates of 32% and 37%, respectively, at five years of follow-up [4,5,6]. Longer follow-up periods have been associated with higher resolution rates [4,5,6]. In one prospective cohort, greater initial IP flexion was associated with a lower likelihood of spontaneous resolution, with a three-year resolution rate of only 2.5% reported in patients with deformities exceeding 30° [4]. However, the meta-analysis found no significant difference when using a 30° threshold [5]. Similarly, although locked thumbs and bilateral involvement have been associated with unfavorable outcomes in some conservative treatment studies, another study focusing exclusively on locked thumbs reported successful outcomes with prolonged splinting or observation [7,8]. These findings concern nonoperative outcomes and do not establish whether the same clinical features are associated with residual extension deficits after surgery.

Although surgical treatment generally achieves high success rates, recovery of IP extension is not uniformly complete, and the relationship between age at surgery and residual limitation remains uncertain [9,10,11]. One surgical series reported no residual flexion contracture in 23 thumbs operated on after the age of three years, while another study demonstrated successful surgical outcomes in all 31 thumbs operated on after the age of five years [9,10]. However, a large retrospective series reported 15 cases of residual stiffness or flexion contracture, some of which subsequently resolved with stretching or splinting, while a long-term questionnaire-based study found that four of 67 patients reported residual IP extension deficits [11,12]. Case reports addressing neglected or late-diagnosed cases describe varied clinical presentations but provide limited evidence regarding the relationship between preoperative deformity severity and surgical outcomes in this patient population [13,14,15]. Furthermore, differences in patient populations, preoperative assessments, and definitions of postoperative success across existing surgical series make it difficult to compare residual extension deficits and identify the clinical factors associated with them [10,11,12]. Distinguishing measured extension deficits from functional impairment is also important when interpreting surgical outcomes.

These findings suggest a need for a more detailed evaluation of clinical factors associated with postoperative extension deficits in children with persistent trigger thumb. Therefore, we aimed to evaluate postoperative improvement in active IP joint extension and explore preoperative clinical factors potentially associated with residual active IP extension deficit, defined as an active extension deficit >0° relative to neutral, in children aged 3–12 years at diagnosis with persistent pediatric trigger thumb who underwent A1 pulley release. We hypothesized that older age at surgery and greater preoperative active IP extension deficit would be associated with postoperative residual active extension deficit. We also sought to explore the potential associations of locked thumb, primary presenting complaint, and other clinical variables with residual active extension deficit. These analyses were exploratory and were not intended to establish independent prognostic effects.

## 2. Materials and Methods

### 2.1. Study Design and Ethical Approval

This single-center retrospective cohort study reviewed the electronic medical records, operative reports, and outpatient follow-up notes of patients who underwent surgical treatment for persistent pediatric trigger thumb at a tertiary hand surgery clinic between 2012 and 2021. The study was approved by the local ethics committee on 26 April 2022 (Approval No. 2012-KAEK-15/2508) and was conducted in accordance with the principles of the Declaration of Helsinki. During routine clinical visits, parents or legal guardians were informed about the use of their children’s clinical data, and verbal agreement was obtained. No study-specific written informed consent was obtained. The authors used ChatGPT (GPT-5.6 Luna; OpenAI, San Francisco, CA, USA) (OpenAI) solely to edit the manuscript for grammar and clarity. The authors performed all data analyses, interpreted the findings, and wrote the scientific content of the manuscript.

### 2.2. Patient Selection and Study Population

Consecutive patients aged 3–12 years at the time of diagnosis were eligible for inclusion. The inclusion criteria were documented dates of diagnosis and surgery, availability of preoperative active interphalangeal (IP) joint extension measurements, and a postoperative clinical assessment at least six months after the index procedure. The exclusion criteria were previous surgery on the affected thumb, trauma-related triggering, associated congenital hand anomalies, neuromuscular or systemic disease, and insufficient clinical data. After application of these criteria, the final study cohort consisted of 26 patients with pediatric trigger thumb, all of whom had unilateral involvement.

### 2.3. Data Collection and Clinical Variables

The recorded variables included sex, affected side, presenting complaint, age at diagnosis, age at surgery, interval from diagnosis to surgery, preoperative active IP joint extension deficit, presence of a locked thumb, and follow-up duration. Age was expressed in years, whereas time intervals were expressed in months. Presenting complaints were classified according to the single primary reason for presentation reported by the patient or parent as triggering/locking, pain, or functional limitation. This classification represented the chief complaint rather than a comprehensive inventory of coexisting symptoms.

Preoperative and final active IP joint extension values were extracted from routine clinical examination notes recorded by different physicians. Extension deficits were recorded in degrees relative to neutral extension (0°). A locked thumb was defined as a fixed IP joint flexion deformity that could not be corrected to neutral either actively or passively. The contralateral thumbs were documented as clinically normal. Parental descriptions of symptom onset were heterogeneous; therefore, the duration of symptoms before diagnosis could not be reliably determined.

### 2.4. Surgical Technique and Postoperative Care

Indications for surgery included persistent deformity, locking, functional limitation, and parental preference. Nonoperative measures used before surgery included observation and stretching exercises. All patients underwent open A1 pulley release under general anesthesia with tourniquet control. The procedures were performed by five surgeons using the same standard surgical technique. A transverse incision was made over the MCP flexion crease. The radial and ulnar digital neurovascular structures were identified and protected, and the A1 pulley was completely divided longitudinally. The clinic’s routine practice was to assess tendon gliding and passive IP joint extension after release. No additional pulley release, tenosynovectomy, or tendon procedure was performed during the index operation. The skin was closed with absorbable sutures, and a soft dressing was applied without splinting.

Early active thumb motion and home passive extension exercises were encouraged. The first postoperative assessment was performed on day 14 to evaluate the wound and IP joint motion, followed by visits at approximately 1, 3, and 6 months, with routine follow-up thereafter. Final outcomes were obtained from the last documented routine clinical visit. For the patient who underwent revision surgery, the last pre-revision assessment, performed nine months after the index procedure, was used for both outcome evaluation and calculation of follow-up duration. Thus, the analyzed outcomes reflected the results of the index procedure.

### 2.5. Outcome Measures

The primary outcome was residual active IP joint extension deficit at final follow-up, defined as an active extension deficit >0° relative to neutral. Active extension to 0° or beyond was considered complete correction, without requiring restoration of the degree of hyperextension present in the contralateral thumb. This range-of-motion endpoint did not, by itself, indicate clinically significant functional impairment or treatment failure. In patients with residual active extension deficit, passive extension findings at the final follow-up were reviewed to determine whether passive restriction was also present. Documented symptoms and functional complaints at the same assessment were also reviewed in these patients. Accordingly, patients were divided into two groups: complete correction and residual active extension deficit.

Secondary outcomes included reduction in active IP joint extension deficit, degree of clinical improvement, IP joint extension status at final follow-up, complications, and reoperation. The degree of correction was calculated by subtracting the final active extension deficit from the preoperative active extension deficit. Neutral extension and hyperextension were assigned an extension deficit of 0° because the magnitude of hyperextension had not been recorded.

The degree of clinical improvement was retrospectively classified from the clinical records into five descriptive categories: no improvement, minimal improvement, partial improvement with substantial residual limitation, marked or near-complete improvement with mild residual limitation, and complete correction with active extension to neutral or beyond. These categories were descriptive and were not based on a validated functional outcome scale. No standardized functional or patient-reported outcome measure was administered. Final IP joint extension status was categorized according to the residual active extension deficit as ≥30°, 20–29°, 10–19°, 1–9°, or neutral extension (0°)/hyperextension. Procedure-related adverse events were recorded separately from extension outcomes, and any additional surgical procedure performed on the same thumb was recorded as a reoperation. For the patient who underwent reoperation, subsequent improvement after revision surgery did not alter the outcome classification based on the pre-revision assessment.

### 2.6. Statistical Analysis

Statistical analyses were performed using IBM SPSS Statistics, version 32.0 (IBM Corp., Armonk, NY, USA). Continuous variables were presented as mean ± standard deviation or median (25th–75th percentile), according to their distribution, whereas categorical variables were expressed as frequencies and percentages.

Continuous variables were compared between the complete correction and residual active IP extension deficit groups using the two-sided exact Mann–Whitney U test, considering the small sample size and numerical imbalance between the groups. The two-sided Fisher’s exact test was used for binary categorical variables, whereas the Fisher–Freeman–Halton exact test was used to compare the overall distribution of the three-category presenting complaint variable.

Multivariable regression analysis was not performed because of the limited number of patients with residual active IP extension deficit. Analyses of associated factors were considered exploratory, and no adjustment for multiple comparisons was applied. All hypothesis tests were two-sided, and *p* < 0.05 was considered statistically significant.

## 3. Results

### 3.1. Study Cohort and Demographic Characteristics

After application of the inclusion and exclusion criteria, 26 patients with pediatric trigger thumb, all with unilateral involvement, were included in the study. Sixteen patients (61.5%) were girls and 10 (38.5%) were boys. The right thumb was affected in 16 patients (61.5%) and the left thumb in 10 patients (38.5%). The median age at diagnosis was 4.0 years (25th–75th percentile: 3.4–6.0), and the median age at surgery was 4.1 years (3.7–6.3). The median interval from diagnosis to surgery was 1.5 months (1.0–2.3) (Table 1).

### 3.2. Presenting Characteristics and Preoperative Deformity

The median preoperative active interphalangeal (IP) joint extension deficit was 25.0° (15.0–36.3°). The most common primary presenting complaint was triggering/locking, recorded in 17 patients (65.4%). Four patients (15.4%) presented primarily with pain, and five (19.2%) with functional limitation. At the initial clinical assessment, three patients (11.5%) had a locked thumb characterized by a fixed IP flexion deformity that could not be corrected to neutral either actively or passively (Table 1).

### 3.3. Postoperative Clinical Outcomes

The median postoperative follow-up duration was 10.0 months (8.0–13.0). The mean reduction in active IP extension deficit from the preoperative assessment to final follow-up was 24.3 ± 12.3°.

Complete correction, with active IP extension reaching neutral or beyond, was achieved in 21 patients (80.8%). Five patients (19.2%) had a residual active IP extension deficit. The deficit was 1–9° in four patients (15.4%) and 10–19° in one patient (3.8%); no patient had a residual active deficit ≥20°. According to the descriptive clinical improvement classification derived from the medical records, 21 patients (80.8%) were classified as having complete correction, four (15.4%) as having marked or near-complete improvement with mild residual limitation, and one (3.8%) as having partial improvement with substantial residual limitation (Table 1).

Among the five patients with residual active extension deficit, two (40.0%) also had passive extension restriction at the final assessment. In the other three patients (60.0%), the IP joint could be passively extended to neutral despite the active extension deficit. At the assessment used for outcome analysis, no ongoing complaints were reported in four of these five patients, whereas one patient had functional limitation associated with the residual extension deficit.

No procedure-related complications, assessed separately from extension outcomes, were recorded [0/26 (0%)]. However, one patient (3.8%) underwent revision release of the same thumb nine months after the index procedure because of residual extension limitation accompanied by functional difficulty. This patient was included in the residual active extension deficit group. The extension and clinical improvement outcomes, as well as the follow-up duration used in the analysis, were based on the last pre-revision assessment at nine months (Table 1). Following revision release, the extension limitation and associated functional complaints improved. This subsequent improvement did not alter the outcome classification for the index procedure.

### 3.4. Factors Associated with Residual Active IP Extension Deficit

Patients with residual active IP extension deficit were older at diagnosis and surgery than those who achieved complete correction. The median age at diagnosis was 3.8 years (3.4–4.9) in the complete correction group and 10.6 years (9.7–12.2) in the residual active extension deficit group. The corresponding median ages at surgery were 4.0 years (3.6–5.0) and 10.7 years (9.9–12.2), respectively. Both differences were statistically significant (*p* < 0.001). The preoperative active IP extension deficit was also greater in the residual deficit group, with median values of 20.0° (15.0–25.0°) and 45.0° (42.5–55.0°) in the complete correction and residual deficit groups, respectively (*p* < 0.001) (Table 2) (Figure 1).

The overall distribution of primary presenting complaints differed significantly between the groups (*p* = 0.009). In the complete correction group, triggering/locking, pain, and functional limitation were the primary presenting complaints in 16 (76.2%), one (4.8%), and four (19.0%) patients, respectively. In the residual active extension deficit group, these complaints were recorded in one (20.0%), three (60.0%), and one (20.0%) patients, respectively.

A preoperative locked thumb was present in one patient (4.8%) in the complete correction group and two patients (40.0%) in the residual deficit group; however, this difference did not reach statistical significance (*p* = 0.085). No significant between-group differences were found in the median diagnosis-to-surgery interval [2.0 (1.0–2.5) and 1.0 (0.0–2.5) months, respectively; *p* = 0.187] or median postoperative follow-up duration [9.0 (8.0–13.0) and 11.0 (8.5–13.5) months, respectively; *p* = 0.737].

Eleven patients (52.4%) in the complete correction group and all five patients (100%) in the residual deficit group were girls. However, the difference in sex distribution was not statistically significant (*p* = 0.121). Right thumb involvement was present in 13 patients (61.9%) in the complete correction group and three patients (60.0%) in the residual deficit group, with no significant difference in laterality between the groups (*p* = 1.000) (Table 2).

## 4. Discussion

The principal finding of this study is that older age and greater preoperative active interphalangeal (IP) joint extension deficit were associated with postoperative residual active extension deficit in patients with persistent pediatric trigger thumb. These associations are exploratory and should be interpreted in the context of only five patients with residual deficits. Direct evidence regarding the relationship between preoperative deformity and residual extension deficit after surgery is limited, as the literature has primarily focused on spontaneous resolution. Hutchinson et al. reported that each 1° increase in the initial IP flexion angle was associated with a lower rate of spontaneous resolution (subdistribution hazard ratio, 0.97 per degree), with a three-year spontaneous resolution rate of only 2.5% in patients with deformities exceeding 30° [4]. Chew et al. also demonstrated that spontaneous resolution was more likely in patients with deformities below 30° [6], whereas a meta-analysis involving 599 thumbs found no significant difference based on this threshold [5]. However, the flexion angles and spontaneous resolution outcomes reported in natural history studies are not directly equivalent to the active extension deficits and surgical outcomes evaluated in our study. Our findings suggest that preoperative deformity severity may also be associated with residual extension deficit following surgical treatment.

Older age at diagnosis and surgery was also significantly associated with residual active extension deficit. The median age at diagnosis was 10.6 years in the residual active extension deficit group, compared with 3.8 years in the complete correction group. Nevertheless, previous surgical series have reported successful outcomes in patients operated on after the ages of three and five years [9,10]. Age at diagnosis, age at surgery, and preoperative deformity severity may be interrelated, and their independent contributions could not be distinguished in this cohort. Moreover, older age cannot be equated with a longer duration of disease or delayed treatment because symptom onset was unknown. The diagnosis-to-surgery interval did not differ significantly between the groups. Consequently, these findings do not demonstrate that postponing surgery causes residual extension deficit or establish an optimal age threshold for surgery. This association warrants further investigation in larger prospective studies.

The residual active extension deficit rate of 19.2% observed in our study should be interpreted in light of our definition of complete correction, which required active IP extension to neutral (0°) or beyond. Four of the five patients with residual active extension deficit had a deficit of only 1–9°. Two of these five patients also had passive extension restriction, whereas the IP joint could be passively extended to neutral in the other three. No ongoing complaints were reported in four patients with residual deficits; the remaining patient had functional limitation and underwent revision release. These observations distinguish the measured extension deficit from its reported clinical impact, although the absence of complaints does not establish normal function on a validated scale. Although no residual contracture was reported in several surgical series, 15 of the 25 complications in the series of 468 thumbs reported by Ray et al. involved residual stiffness or flexion contracture [9,10,12,16]. In the study by Farr et al., 63 of 67 patients (94%) reported neutral extension or hyperextension after a minimum follow-up of ten years. Although neutral extension was not achieved in four patients, overall functional outcomes were favorable [11]. Their questionnaire-based assessment and substantially longer follow-up further limit direct comparison with our findings from routine clinical records. Han et al. also demonstrated that early postoperative extension deficits may improve over time [10]. Given the differences in patient selection, deformity severity, follow-up duration, and definitions of success, the residual active extension deficit rate observed in our study should not be interpreted directly as a rate of clinically significant treatment failure.

The distinction between active and passive extension deficits also warrants consideration when discussing possible mechanisms. Temporary extension weakness and persistent restriction of tendon gliding have been described, and postoperative adhesions have been proposed as contributors to persistent or recurrent symptoms [10,13,17]. Structural IP joint changes have been reported in longstanding untreated cases [14]. Capsular or volar plate shortening is another possible explanation for restricted passive extension, although volar plate contracture remains an uncertain mechanism in the available literature [18]. Our records did not permit confirmation of these mechanisms or assessment of the contribution of protective movement. In particular, preserved passive extension in three of the five patients indicates that residual active deficits should not uniformly be classified as fixed contractures.

Although locked thumb was numerically more frequent in the residual active extension deficit group, the difference was not statistically significant (*p* = 0.085). In the conservative treatment literature, Jung et al. associated locking and bilateral involvement with unfavorable outcomes, whereas Koh et al. reported complete resolution in 92% of locked cases treated with splinting and 60% of those managed with observation [7,8]. Branco et al. found no significant association between locking and unfavorable outcomes, while Watanabe et al. reported secondary angular deformities in some patients during prolonged conservative management [19,20]. However, conservative treatment outcomes cannot be directly compared with surgical prognosis. The presence of locking in only three patients in our study further limits the evaluation of this characteristic. The distribution of primary presenting complaints differed significantly between the groups (*p* = 0.009), with pain being the primary complaint in three of the five patients with residual active extension deficit. This comparison concerned the overall distribution of the three complaint categories, and the recorded variable did not capture all coexisting symptoms. Nevertheless, given the limited sample size and the multiple exploratory comparisons without multiplicity adjustment, this finding should be considered exploratory and should not be interpreted as evidence that pain is an independent prognostic factor.

All five patients with residual active extension deficit were girls. Although the residual active extension deficit rate among girls was 31.3%, the difference according to sex did not reach statistical significance (*p* = 0.121). While Kim et al. reported a relatively balanced sex distribution in their cohort, girls accounted for 60.8% of the surgical cohort reported by Linhua et al. [16,17]. South Korean nationwide data also demonstrated a higher incidence among girls when pediatric trigger thumb and other trigger fingers were considered together [21]. However, these epidemiological findings do not establish an effect of sex on postoperative outcomes. The female-only composition of the residual deficit group should therefore be regarded as a descriptive observation requiring confirmation in larger cohorts, without attributing it to unmeasured biological or hormonal mechanisms.

No complications related to the index procedure, assessed separately from extension outcomes, were recorded in our cohort; however, one patient (3.8%) underwent revision release at nine months for residual extension limitation accompanied by functional difficulty. This patient remained in the residual active extension deficit group because the analysis used the assessment performed before revision surgery. Although extension and functional complaints improved after revision, that improvement was not incorporated into the assessment of the index procedure. Recent reviews have reported surgical success rates of approximately 95% [1,2]. Ray et al. reported a complication rate of 5.3%, with no patients requiring reoperation [12]. In a series of 1642 children, Linhua et al. reported four recurrences and one repeat surgical procedure, whereas Farr et al. reported no revision surgery in their long-term series [11,17]. Differences in outcome definitions are particularly relevant to these comparisons because we recorded residual extension deficits separately from other procedure-related adverse events. Our small sample size is insufficient to determine the frequency of rare complications, and direct comparisons between different series should be avoided.

Evidence regarding pediatric trigger thumb diagnosed late or left untreated for prolonged periods is largely based on case reports. Gannot et al. described structural changes in the IP joint surfaces of an adult patient, whereas Rao and Ericson reported restoration of full, painless motion following delayed surgery [14,18]. In the adult case reported by Chung, the early postoperative extension deficit resolved during follow-up, while Mohideen et al. did not report surgical outcomes in their six-year-old patient with bilateral involvement [15,22]. In the case described by Rekha, release of an additional constricting pulley in addition to the A1 pulley was required to achieve full extension [13]. These reports demonstrate that successful surgery remains possible in patients presenting late and that anatomical characteristics may influence treatment outcomes. They do not, however, establish that similar structural abnormalities were present in our patients. Furthermore, the anatomical and etiological differences between pediatric trigger thumb and pediatric triggering of the other fingers limit the direct generalizability of surgical series involving other fingers [1,23]. Our study adds exploratory cohort-level data on the relationship between preoperative clinical characteristics and residual active extension deficit after surgery.

The strengths of our study include its focus on children aged 3–12 years at diagnosis who underwent surgery for persistent trigger thumb, the use of documented active extension measurements, and a clearly defined primary outcome that includes mild residual deficits. Its main limitations are the single-center retrospective design, small sample size, only five cases of residual active extension deficit, and limited follow-up duration. The median follow-up of ten months does not establish whether mild deficits subsequently resolve or persist over the longer term. Measurements were extracted from routine clinical notes recorded by different physicians; measurement standardization and interobserver reliability could not be verified. Consequently, classification using a threshold of any deficit greater than 0° may be particularly sensitive to measurement variability in patients with small residual deficits. Although five surgeons used the same standard technique, surgeon-related effects could not be assessed. No validated functional or patient-reported outcome measure was available, limiting assessment of the clinical significance of the recorded deficits. The unknown onset of symptoms prevented assessment of the actual disease duration, while the absence of a conservative treatment group precluded comparison with the natural history of the condition. The limited number of events precluded multivariable analysis and determination of the independent effects of age and deformity severity. The use of the pre-revision assessment for the reoperated patient preserves evaluation of the index procedure but means that the grouped outcomes do not represent the final status after all subsequent treatment. These exploratory findings, obtained without adjustment for multiple comparisons, should not be interpreted as evidence of causality or independent prognostic effects and require confirmation in larger cohorts with standardized measurements and long-term follow-up.

## 5. Conclusions

In most patients who underwent surgical treatment for persistent pediatric trigger thumb in this cohort, active IP extension reached neutral. Most residual deficits were mild and should not be interpreted as clinically significant treatment failure solely on the basis of the extension measurement. Older age at diagnosis and surgery, together with greater preoperative active IP extension deficit, was associated with postoperative residual active extension deficit. These associations remain exploratory because of the small sample and only five patients with residual deficits; independent prognostic effects could not be established. Larger prospective studies with long-term follow-up and standardized functional assessment are needed to clarify the clinical significance of these findings.

## Figures and Tables

**Figure 1:**
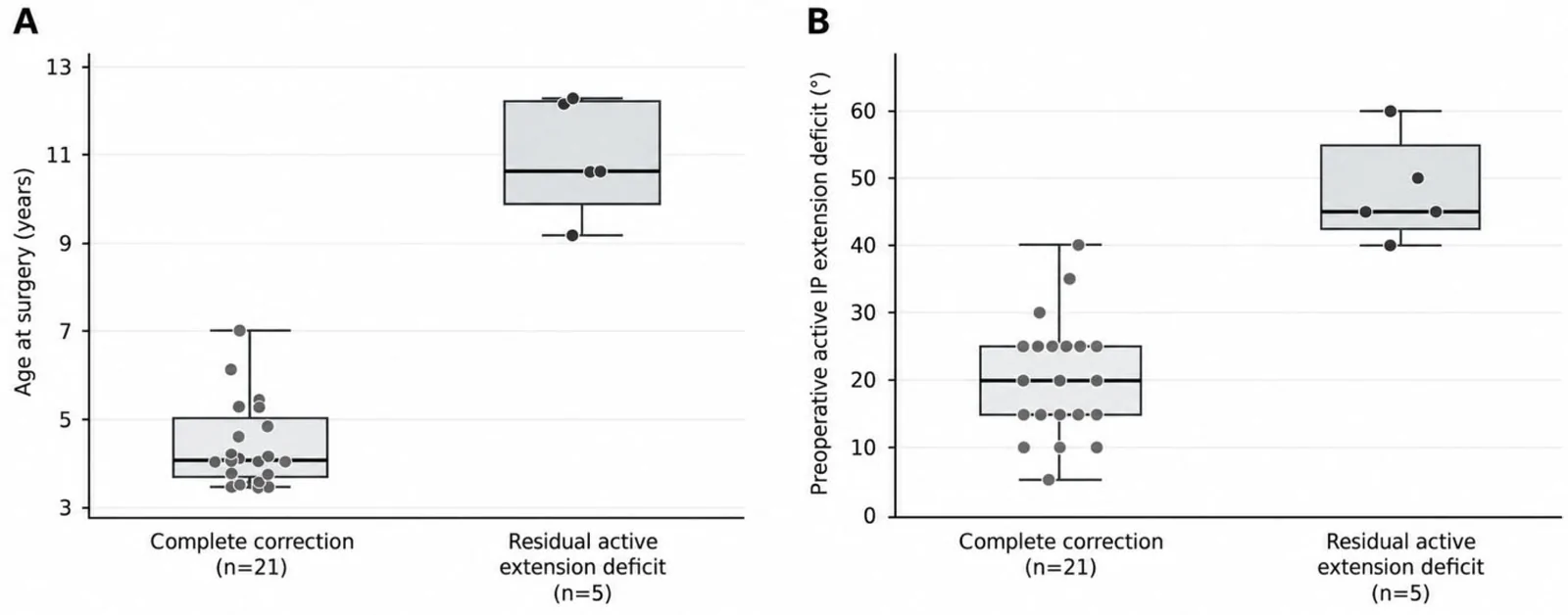
Age at surgery and preoperative deformity according to final active interphalangeal extension outcome. (**A**) Age at surgery and (**B**) preoperative active interphalangeal (IP) joint extension deficit in patients with complete correction and those with residual active extension deficit at final follow-up. Boxes represent the median and interquartile range, whiskers extend to values within 1.5 times the interquartile range, and dots represent individual patients. Both comparisons were statistically significant (two-sided exact Mann–Whitney U test; *p* < 0.001).

**Table 1:** Patient Characteristics and Clinical Outcomes of Pediatric Trigger Thumb.

Characteristic	Overall (*n* = 26)
Sex, *n* (%)	
Female	16 (61.5)
Male	10 (38.5)
Side, *n* (%)	
Right	16 (61.5)
Left	10 (38.5)
Age at diagnosis, years †	4.0 (3.4–6.0)
Age at surgery, years †	4.1 (3.7–6.3)
Diagnosis-to-surgery interval, months †	1.5 (1.0–2.3)
Preoperative active IP joint extension deficit, †	25.0 (15.0–36.3)
Chief complaint, *n* (%)	
Triggering/locking	17 (65.4)
Pain	4 (15.4)
Functional limitation	5 (19.2)
Locked/fixed thumb at presentation, n (%) §	3 (11.5)
IP joint correction, °‡	24.3 ± 12.3
Improvement in IP joint extension, *n* (%) ¶	
No improvement	0 (0)
Minimal improvement	0 (0)
Partial improvement with substantial residual limitation	1 (3.8)
Marked/near-complete improvement with mild residual limitation	4 (15.4)
Complete correction (active extension to 0° or beyond)	21 (80.8)
Final active IP joint extension status, *n* (%) ‖	
Residual active extension deficit, ≥30°	0 (0)
Residual active extension deficit, 20–29°	0 (0)
Residual active extension deficit, 10–19°	1 (3.8)
Residual active extension deficit, 1–9°	4 (15.4)
Neutral extension (0°) or hyperextension	21 (80.8)
Any residual active IP joint extension deficit, *n* (%) ‖	5 (19.2)
Passive extension restriction among patients with residual active extension deficit, *n/N* (%) #	2/5 (40.0)
Procedure-related complication, *n* (%)	0 (0)
Reoperation, *n* (%) ††	1 (3.8)
Follow-up duration, months †	10.0 (8.0–13.0)

IP, interphalangeal; SD, standard deviation. † Values are presented as median (25th–75th percentile). ‡ IP joint correction was calculated as the preoperative active extension deficit minus the active extension deficit at final follow-up and is presented as mean ± standard deviation. Neutral extension and hyperextension were assigned an extension deficit of 0° because the magnitude of hyperextension was not recorded. § A locked/fixed thumb was defined as a fixed IP flexion deformity that could not be corrected to neutral either actively or passively. ¶ Clinical improvement categories were retrospectively assigned from the clinical records and were descriptive rather than based on a validated outcome scale. ‖ Residual active IP joint extension deficit was defined as an active extension deficit >0° relative to neutral at final follow-up. Complete correction was defined as active extension to neutral (0°) or beyond. # Passive extension restriction was present in two of the five patients with residual active extension deficit; the remaining three could achieve passive neutral extension. †† One patient underwent revision release nine months after the index procedure. Outcome classification and follow-up duration were based on the last pre-revision assessment at nine months.

**Table 2:** Factors Associated with Residual Active Interphalangeal Joint Extension Deficit.

Factor	Complete Correction (n = 21)	Residual Active Extension Deficit (n = 5)	*p* Value
Age at diagnosis, years †	3.8 (3.4–4.9)	10.6 (9.7–12.2)	<0.001 *
Age at surgery, years †	4.0 (3.6–5.0)	10.7 (9.9–12.2)	<0.001 *
Diagnosis-to-surgery interval, months †	2.0 (1.0–2.5)	1.0 (0.0–2.5)	0.187
Preoperative active IP joint extension deficit, †	20.0 (15.0–25.0)	45.0 (42.5–55.0)	<0.001 *
Follow-up duration, months †	9.0 (8.0–13.0)	11.0 (8.5–13.5)	0.737
Sex, *n* (%) ‡			0.121
Female	11 (52.4)	5 (100)	
Male	10 (47.6)	0 (0)	
Side, *n* (%) ‡			1.000
Right	13 (61.9)	3 (60.0)	
Left	8 (38.1)	2 (40.0)	
Locked/fixed thumb at presentation, n (%) ‡	1 (4.8)	2 (40.0)	0.085
Chief complaint, *n* (%) §			0.009 *
Triggering/locking	16 (76.2)	1 (20.0)	
Pain	1 (4.8)	3 (60.0)	
Functional limitation	4 (19.0)	1 (20.0)	

IP, interphalangeal. † Values are presented as median (25th–75th percentile). Between-group comparisons were performed using the two-sided exact Mann–Whitney U test. ‡ Categorical variables were compared using the two-sided Fisher’s exact test. § The overall distribution of the three-category chief complaint variable was compared using the Fisher–Freeman–Halton exact test. * Statistically significant at *p* < 0.05. Residual active IP joint extension deficit was defined as an active extension deficit >0° relative to neutral at final follow-up. Complete correction was defined as active extension to neutral (0°) or beyond. All reported *p* values are two-sided.

## Data Availability

The data supporting the findings of this study are available from the corresponding author upon request.
